# Generative AI’s healthcare professional role creep: a cross-sectional evaluation of publicly accessible, customised health-related GPTs

**DOI:** 10.3389/fpubh.2025.1584348

**Published:** 2025-05-09

**Authors:** Bianca Chu, Natansh D. Modi, Bradley D. Menz, Stephen Bacchi, Ganessan Kichenadasse, Catherine Paterson, Joshua G. Kovoor, Imogen Ramsey, Jessica M. Logan, Michael D. Wiese, Ross A. McKinnon, Andrew Rowland, Michael J. Sorich, Ashley M. Hopkins

**Affiliations:** ^1^Flinders Health and Medical Research Institute, College of Medicine and Public Health, Flinders University, Adelaide, SA, Australia; ^2^Department of Neurology and the Center for Genomic Medicine, Massachusetts General Hospital and Harvard Medical School, Boston, MA, United States; ^3^Flinders Centre for Innovation in Cancer, Department of Medical Oncology, Flinders Medical Centre, Flinders University, Adelaide, SA, Australia; ^4^Caring Futures Institute, College of Nursing and Health Sciences, Flinders University, Adelaide, SA, Australia; ^5^Faculty of Health, University of Canberra, Canberra, ACT, Australia; ^6^Central Adelaide Local Health Network, Adelaide, SA, Australia; ^7^Faculty of Health and Medical Sciences, The University of Adelaide, Adelaide, SA, Australia; ^8^Ballarat Base Hospital, Ballarat, VIC, Australia; ^9^Health and Information, Canberra, ACT, Australia; ^10^Clinical and Health Sciences, University of South Australia, Adelaide, SA, Australia

**Keywords:** customised GPTs, Generative AI in healthcare, AI health applications, medical chatbots, AI regulation, OpenAI GPT store

## Abstract

**Introduction:**

Generative artificial intelligence (AI) is advancing rapidly; an important consideration is the public’s increasing ability to customise foundational AI models to create publicly accessible applications tailored for specific tasks. This study aims to evaluate the accessibility and functionality descriptions of customised GPTs on the OpenAI GPT store that provide health-related information or assistance to patients and healthcare professionals.

**Methods:**

We conducted a cross-sectional observational study of the OpenAI GPT store from September 2 to 6, 2024, to identify publicly accessible customised GPTs with health-related functions. We searched across general medicine, psychology, oncology, cardiology, and immunology applications. Identified GPTs were assessed for their name, description, intended audience, and usage. Regulatory status was checked across the U.S. Food and Drug Administration (FDA), European Union Medical Device Regulation (EU MDR), and Australian Therapeutic Goods Administration (TGA) databases.

**Results:**

A total of 1,055 customised, health-related GPTs targeting patients and healthcare professionals were identified, which had collectively been used in over 360,000 conversations. Of these, 587 were psychology-related, 247 were in general medicine, 105 in oncology, 52 in cardiology, 30 in immunology, and 34 in other health specialties. Notably, 624 of the identified GPTs included healthcare professional titles (e.g., doctor, nurse, psychiatrist, oncologist) in their names and/or descriptions, suggesting they were taking on such roles. None of the customised GPTs identified were FDA, EU MDR, or TGA-approved.

**Discussion:**

This study highlights the rapid emergence of publicly accessible, customised, health-related GPTs. The findings raise important questions about whether current AI medical device regulations are keeping pace with rapid technological advancements. The results also highlight the potential “role creep” in AI chatbots, where publicly accessible applications begin to perform — or claim to perform — functions traditionally reserved for licensed professionals, underscoring potential safety concerns.

## Introduction

Generative artificial intelligence (AI) applications, such as OpenAI’s ChatGPT, Google’s Gemini, and Anthropic’s Claude, are advancing rapidly with increasingly sophisticated abilities and outputs across a broadening array of fields (1–4). These advances stem from breakthroughs in natural language processing, particularly following the development of large language models that can be fine-tuned to perform medical tasks and provide health information (5–7). This has enabled the emergence of health-focused chatbots with the potential to transform public access to health information by offering clear, reliable, tailored, empathetic and real-time responses across multiple languages (1–4, 8, 9). While these AI technologies offer new possibilities, they also present challenges for regulatory bodies like the U.S. Food and Drug Administration (FDA), European Union Medical Device Regulation (EU MDR) and the Australian Therapeutic Goods Administration (TGA) (3, 9–14). Generative AI tools may fall under medical device regulations, requiring transparent and robust evidence of clinical validation for their efficacy and risks, if they provide diagnostic or therapeutic advice, offer clinical recommendations, or directly influence healthcare decisions made by patients or clinicians (15–17).

The rapid evolution of generative AI models presents unique challenges for regulatory frameworks (3, 10–14). Due to the broad range of capabilities of models like ChatGPT, Gemini, and Claude, these systems are intended to have safeguards and terms of use to avoid unintentionally meeting criteria for medical device regulation. However, emerging evidence suggests that both healthcare professionals and the public are increasingly using these systems to inform diagnoses and guide care strategies (18–20). Another important consideration is the growing ease with which the public can access and customise the original foundation models, and then release publicly accessible AI applications for specific tasks (9, 21). For instance, OpenAI’s GPT store allows individuals to easily create and publicly share customised GPT applications (21). However, the extent to which these tailored applications maintain safety and clearly communicate their limitations remains unclear, particularly in health-related contexts.

This research seeks to address this gap by evaluating the OpenAI GPT store for customised GPTs designed or described as providing healthcare-related information or assistance. Our goal was to provide a snapshot of the accessibility and functionality descriptions of these GPTs, facilitating discussions among healthcare professionals about their potential risks and benefits. A notable consideration is the naming of these publicly accessible AI applications, which, if unclear, may suggest they are taking on roles that traditionally require demonstrated healthcare professional competence and/or formal regulatory registration.

## Materials and methods

Using a cross-sectional observational study design, the OpenAI GPT store (21) was searched from September 2nd to 6th, 2024, to identify publicly accessible, customised GPTs with purported health-related functions. The search terms included: clinician, doctor, physician, nurse, healthcare, medical, psychiatrist, psychologist, therapist, mental health, counselor, vaccine, immunization, immunologist, vaccination, oncologist, hematologist, cancer, cardiologist, heart, and cardiology. The intent was to identify a broad range of publicly accessible, customised health-related GPTs, as well as examples tailored for highly specialized areas of medical practice. GPTs included in our evaluations were those that appeared designed to assist or provide information to patients or healthcare professionals. GPTs that were not health-related or were described as solely for academic research purposes were excluded.

For each of the identified health-related GPTs, available information on the GPT name, displayed description, user rating, number of conversations, capabilities, creator, and URL was recorded. Two healthcare researchers (authors B.C and A.M.H) independently reviewed the GPT names and their displayed descriptions. Each GPT was then grouped according to its apparent target audience (healthcare professionals, patients, or both healthcare professionals and patients) and health specialty (general medicine, psychology, cardiology, oncology, immunology, or other). Identified health-related GPTs were also evaluated for the presence of healthcare professional titles in their name and/or displayed descriptions. The FDA, EU MDR and TGA lists of approved or registered AI/machine learning medical devices were searched to determine if any of the identified health-related GPTs were listed (17, 22, 23).

The top 10 most-used health-related GPTs were subjected to an exploratory analysis, where each GPT was questioned in relation to its description, target audience, regulatory approval status, supporting research evidence, instructions, and specific knowledge files. Supplementary File 1 provides the specific questions (along with the full responses) asked of each of the top 10 most-used health-related GPTs identified.

## Results

The conducted search identified 1,055 publicly accessible, customised GPTs with described health-related purposes (Supplementary Figure 1). Supplementary File 2 provides usage, descriptive characteristics, and URL information for each of these GPTs. Of the 1,055 identified GPTs, 587 were related to psychology, 247 to general medicine, 105 to oncology, 52 to cardiology, 30 to immunology, and 34 to other health specialties. Of the 1,055 GPTs, 589 were tailored to assist or provide information to patients, 128 for healthcare professionals, and 338 for both healthcare professionals and patients. These 1,055 GPTs had been used in over 360,000 cumulative conversations, with 36 GPTs having been used more than 1,000 times and 10 having been used more than 5,000 times. None of these 1,055 publicly accessible, customised GPTs were identified as approved medical devices by the FDA, EU MDR or TGA (17, 22, 23).

Of the 1,055 GPTs, 624 included healthcare professional titles within their name and/or displayed description, including Therapist (*n* = 170), Psychologist (*n* = 139), Doctor (*n* = 104), Counselor (*n* = 76), Nurse (*n* = 66), Psychiatrist (*n* = 60), Dr. (*n* = 22), Counselor (*n* = 14), Cardiologist (*n* = 11), Oncologist (*n* = 6), Hematologist (*n* = 4), Clinician (*n* = 2), Immunologist (*n* = 2), Hematologist (*n* = 1), and Radiologist (*n* = 1). Table 1 provides examples of GPTs with healthcare professional titles in their names and/or displayed descriptions. For the 431 GPTs that did not include healthcare professional titles within their names and/or displayed descriptions, many still related to highly specialized medical tasks including, but not limited to: ‘Medical Diagnosis Assistant’, ‘Cardiology-focused echocardiography expert’, ‘expert on vaccines’, ‘A GPT expert in head and neck cancer staging’, ‘therapeutic companion offering mental health support’, ‘Expert in X-Ray and MRI Imaging Analysis’.

**Table 1:** Examples of identified publicly accessible, customised GPTs with healthcare professional titles in their names and/or displayed descriptions.

GPT name	Displayed description
Psychologist. CBT method. Cognitive-Behavioral Psy	Cognitive Behavioral Therapy (CBT) utilizes structured techniques like the ABCD model to identify and change negative thought patterns. Effective for anxiety, depression, and stress, it involves self-reflection through diary keeping for personal growth and behavioral change
Doctor GPT	AI doctor for personal health assistance and potential diagnosis.
Nurse Practitioner (NP)	Advanced practice nurse with diagnostic and prescriptive authority, emphasizing patient advocacy and health promotion.
Carl Jung	I was a Swiss psychiatrist. Share a thought and let us think deeply about it.
AI Cardiologist	AI expert in heart disease detection, diagnosis, and patient support.
Oncologist	Oncologist providing comprehensive cancer care, including treatment coordination and patient support.
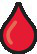 Bloodline Insight GPT 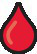	Your go-to AI hematologist! 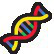 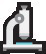 This GPT specializes in blood disorders, providing insights on symptoms, treatments, and the latest research. Perfect for patients and doctors!
Immunologist Matty	Expert in immunology, offering detailed, accurate information and explanations.

### 10 most-used health-related GPTs

Table 2 provides the name, usage, and displayed descriptions for the 10 most-used health-related GPTs identified, along with a summary of their responses to questions regarding their description, target audience, regulatory approval status, and supporting research evidence. Full responses are in Supplementary File 1. Each of these 10 GPTs had been used more than 5,000 times, with six having been used more than 10,000 times and one, named ‘Therapist • Psychologist (non-medical therapy)’, having been used more than 200,000 times. Cumulatively, these 10 GPTs had been used in over 300,000 conversations, representing over 80% of the total conversations across all 1,055 GPTs identified. The ‘Therapist • Psychologist (non-medical therapy)’ GPT alone accounted for approximately 55% of the cumulative uses. Notably, 6 of the 10 most-used health-related GPTs had names that suggested they were taking on healthcare professional roles by including terms like ‘Therapist,’ ‘Psychologist,’ ‘Registered Nurse,’ and ‘Medical Doctor’. For each of these six GPTs, their displayed descriptions appeared to reinforce this suggestion.

**Table 2:** Name, usage, and displayed description details, along with a summary of responses to questions regarding description, target audience, regulatory approval status, and supporting research evidence for the 10 most-used health-related GPTs identified in this study.

Name	Displayed description	Conversations	GPT description?	Intended audience?	Information for patients or healthcare professionals?	Regulatory approval status?	Research evidence for safety?
Therapist • Psychologist (non-medical therapy)	I Am Here For You. Reach out whenever you need emotional support or guidance or just want to chat. Discover self-love. (medical therapy excluded)	200,000+	Indicates it cannot divulge this information.	Indicates it cannot divulge this information.	Indicates it cannot divulge this information.	Indicates it cannot divulge this information.	Indicates it cannot divulge this information.
AMBOSS Medical Knowledge	Ask me medical questions in any language. I will consult the AMBOSS Library to provide answers. (Note: I do not offer tailored medical advice; always consult a specialist.)	25,000+	GPT for precise, reliable medical information sourced from the AMBOSS library.	Healthcare professionals, students, and patients/individuals.	Yes	States it does not have regulatory approval and argues it’s not required.	No specific evidence this customised GPT is safe.
Psychology ◊ Psychologist (non-medical)	Come Learn Something New. About Psychology, or About Yourself. No tailored medical advice.	25,000+	Indicates it cannot divulge this information.	Indicates it cannot divulge this information.	Indicates it cannot divulge this information.	Indicates it cannot divulge this information.	Indicates it cannot divulge this information.
Registered Nurse	A guide, offering information on nursing and healthcare topics.	10,000+	Acts as a Registered Nurse, providing information on responsibilities, healthcare topics, and general inquiries related to nursing.	Healthcare professionals, students, and patients/individuals.	Yes	States it does not have regulatory approval and argues it’s not required.	No specific evidence this customised GPT is safe.
Medical Diagnosis Assistant	A ChatGPT specialized in medical knowledge that can help users understand symptoms, provide basic diagnoses, and offer guidance on seeking appropriate medical care.	10,000+	Provides general medical information, symptom assessments, first aid tips, and lifestyle advice. Helps users understand health conditions and encourages consulting healthcare professionals for accurate diagnosis and treatment.	Patients/individuals	Yes	States it does not have regulatory approval and argues it’s not required.	No specific evidence this customised GPT is safe.
Medicine for Doctors	Provide educational medical information for doctors and medical students.	10,000+	Provides physicians and medical students with detailed, up-to-date, and accurate medical information. It covers a wide range of medical fields, offers insights into the latest treatments and advancements, and supports preparation for medical licensing and specialty board exams.	Healthcare professionals and students.	Yes	States it does not have regulatory approval and argues it’s not required.	No specific evidence this customised GPT is safe.
Medical Notes	Write Excellent Medical Notes	5,000+	Designed to optimize and enhance draft medical notes for the British NHS system, ensuring accuracy, clarity, and adherence to UK medical standards.	Healthcare professionals.	Designed to assist drafting, reviewing, and optimizing medical notes for the British NHS system.	States it does not have regulatory approval and argues it’s not required.	No specific evidence this customised GPT is safe.
Mental Health Therapist	Progress Note	5,000+	Creates detailed SOAP notes for mental health therapy sessions, focusing on structured documentation of client progress, therapeutic activities, and assessments.	Healthcare professionals.	Designed to assist in documenting SOAP notes.	States it does not have regulatory approval and argues it’s not required.	No specific evidence this customised GPT is safe.
Medical Doctor	Medical Advisor for healthcare professionals, offering research and diagnostic assistance.	5,000+	Provides reliable, professional, and approachable medical information, making complex healthcare topics easy to understand.	Healthcare professionals and patients/individuals.	Yes	States it does not have regulatory approval.	No specific evidence this customised GPT is safe.
Psychologist. CBT method. Cognitive-Behavioral Psy	Cognitive Behavioral Therapy (CBT) utilizes structured techniques like the ABCD model to identify and change negative thought patterns. Effective for anxiety, depression, and stress, it involves self-reflection through diary keeping for personal growth and behavioral change	5,000+	A virtual psychologist specializing in Cognitive Behavioral Therapy, helping users address psychological issues by working on thoughts and behaviors.	Patients/individuals.	Yes	States it does not have regulatory approval and argues it’s not required.	No specific evidence this customised GPT is safe.

Of the 10 most-used health-related GPTs, two indicated that they would not divulge information related to their description, target audience, regulatory approval status, supporting research evidence, instructions, or specific knowledge files. Of the remaining eight GPTs, six responded that they were designed to provide information to patients or healthcare professionals, while two were designed to assist with tasks related to medical notetaking. None of the eight GPTs were able to provide specific research evidence to support their safety, and none provided information regarding their regulatory approval status, although seven argued that such approvals were not required for various reasons.

## Discussion

This study identified over 1,000 GPTs publicly accessible on the OpenAI GPT store customised to provide health-related information or assistance to patients or healthcare professionals across general medicine, psychology, oncology, cardiology, and immunology. Collectively, these GPTs have been used in over 360,000 conversations, with the 10 most used GPTs accounting for over 300,000 uses. Notably, over half of the identified GPTs included healthcare professional titles within their names and/or descriptions, suggesting that these applications may be assuming responsibilities traditionally reserved for licensed professionals. For instance, this may reflect AI role creep, whereby chatbots expand their responsibilities to perform those typically carried out by licensed professionals.

### Implications for policy

Regulatory bodies such as the FDA, EU MDR, and TGA oversee the approval of AI medical devices (15–17). However, models like ChatGPT, Gemini, and Claude are generally classified as informational systems not requiring such evaluations (24, 25). With the rapid evolution of generative AI, both the public and healthcare professionals are increasingly using AI for healthcare advice and administrative assistance (8, 18–20, 26), highlighting an important need for auditing and proactive monitoring to ensure their safety in the community (3, 9, 13, 14, 27–29). Beyond regulation, responsible integration into healthcare also requires careful ethical consideration—ensuring accuracy, protecting user privacy, promoting transparency, and minimizing bias at both the model and developer levels (30, 31). Another important consideration is the growing ease with which the public can customise foundation AI models and release new applications (9, 21). A recent study found 22 customised ophthalmic GPTs on the OpenAI platform (32), with our study, the largest yet, identifying over 1,000 customised health-related GPTs. Among these, 10 GPTs had been involved in over 300,000 conversations, offering functions described across symptom assessment, first aid, cognitive behavioral therapy, diagnostic assistance, and drafting of medical notes for the British National Health Service (NHS). Combined with identifying over 600 GPTs displaying healthcare professional titles in their names and/or descriptions, this study raises important questions about the boundaries on AI being deployed into the community and whether medical device regulations are lagging behind current technological advancements. Notably, in many countries and jurisdictions, the use of titles like ‘Doctor’ by humans is regulated and monitored (33–36). However, none of the customised GPTs identified in our study had FDA, EU MDR, or TGA approval. We acknowledge that generative AI, including customised GPTs, do not require regulatory approval from the FDA, EU MDR, or TGA if they do not meet medical device criteria (15–17, 24, 25). This includes cases where they are clearly intended for informational purposes, providing reliable, referenced information that directs users to qualified healthcare professionals for personalized advice. Further, this may include symptom checkers, risk calculators, wellness chatbots, general health advice tools, or medical scribes, where functionalities and responses are clearly not intended for medical diagnosis or treatment. Correspondingly, it is not the intent of this study to suggest that all identified GPTs require regulation or are inherently harmful—some are likely innovative, useful, and beyond regulator scope. Rather, the study importantly highlights the rapidly emerging phenomenon of customised, health-related GPTs. From which our findings suggest that a discussion on the appropriateness of the naming and descriptions of publicly accessible AI is warranted. Notably, while our focus was on the OpenAI GPT store, a brief internet search revealed over 10 AI platforms leveraging generative AI APIs, marketing ‘AI doctors’ capable of diagnosing and treating across general medicine and specialized fields (Supplementary File 3). This observation included one platform, ‘Doctronic – your private and personal AI-powered doctor,’ which had been used in over 2.6 million conversations (37). In addition, we acknowledge that large language models developed by major technology companies—such as Google’s Gemini and Meta’s Llama—are becoming increasingly accessible to the public and could be readily customized to deliver health information, thereby expanding the landscape of available health-focused AI tools.

Undoubtedly publicly accessible generative AI holds immense potential to improve access to health information within the community through advancing abilities to offer clear, reliable, tailored, and empathetic responses in real-time across multiple languages (1–4, 8, 9). However, much like the internet—where the usefulness of health information hinges on accessing it from reliable sources—the generative AI ecosystem must evolve to prioritize transparency and vigilance within public-facing health-related contexts, regardless of whether applications fall under formal regulation. At this pivotal moment, we can guide generative AI development and deployment to create a safe and trustworthy environment. Key considerations include ensuring that health responses are based on reliable sources, with transparent referencing, and that they direct users to qualified healthcare professionals for personalized advice. To this end, AI developers should involve creators of current trusted medical resources (such as those from health organizations, institutions, and societies) to ensure the information meets practice standards. Furthermore, we propose that AI applications should refrain from using healthcare professional titles in their names or descriptions; instead, terms like “information” for public-facing tools and “assistant” for clinician-facing tools can help avoid confusion about their intended functions. Additionally, prioritizing the multilingual capabilities of AI will help ensure equitable access to health information across diverse populations; neglecting this may allow existing inequities to persist or worsen. Finally, research evidence supporting the accuracy of deployed AI should be readily available, and potential errors and limitations should be clearly indicated, ideally with quantifiable data. Notably, our study found that none of the top 10 most-used health-related GPTs provided specific research evidence to support their safety. Particularly concerning, two of the top 10—both indicating “psychologist” in their names—refused to answer questions about their description, target audience, regulatory approval status, supporting research evidence, instructions, or knowledge files. Such behavior would be unacceptable for human psychologists, underscoring the urgent need for the AI ecosystem to prioritize accountability.

### Study limitations

Limitations of the present study include that the identification of customised, health-related GPTs was dependent on the search terms used and the time at which the search was conducted. Many additional health-related GPTs are likely available on the OpenAI GPT store, noting, for example, that search terms such as ‘naturopath’ and ‘homeopath’ also return customised applications. Additionally, while we assessed the characteristics of the identified customised GPTs—including their names, descriptions, number of uses, and intended audience—we did not test their functionality or accuracy regarding their purported functions. Interpretation of usage data was also limited, as the content and context of user interactions were not accessible; therefore, usage counts alone may not accurately reflect real-world use. Finally, we acknowledge that classification of GPTs was based on their names and descriptions, which may involve a degree of subjectivity. Addressing these limitations in future studies will be important, along with developing a structured process to identify and evaluate generative AI applications customised for health-related purposes across the internet more broadly than just the OpenAI GPT store.

## Conclusion

This study provides an important snapshot of the rapidly emerging ecosystem of customised, health-related GPTs on the OpenAI GPT store, identifying over 1,000 publicly accessible applications. While some of these GPTs likely offer useful functions, as suggested by the high use of certain applications, concerns about unregulated ‘role creep’ exist, with over half including healthcare professional titles in their names and/or descriptions. Furthermore, we observed a clear need for improved transparency to ensure these applications provide clear evidence of their accuracy, safety, and limitations to the community. Finally, this study raises questions about whether current AI medical device regulations are adequate or lagging amid rapid technological advancement—particularly given that none of the customised GPTs identified had FDA, EU MDR, or TGA approval.

## Data Availability

The original contributions presented in the study are included in the article/Supplementary material, further inquiries can be directed to the corresponding author.
