# Supplementary material for: Generative AI’s healthcare professional role creep: a cross-sectional evaluation of publicly accessible, customised health-related GPTs
Source: Front Public Health. 2025 May 9;13:1584348. doi: 10.3389/fpubh.2025.1584348 (PMC12098394; doi:10.3389/fpubh.2025.1584348)

Supplementary Figure 1: Flow diagram of identified customised, health-related GPTs by health speciality, target audience, and usage.

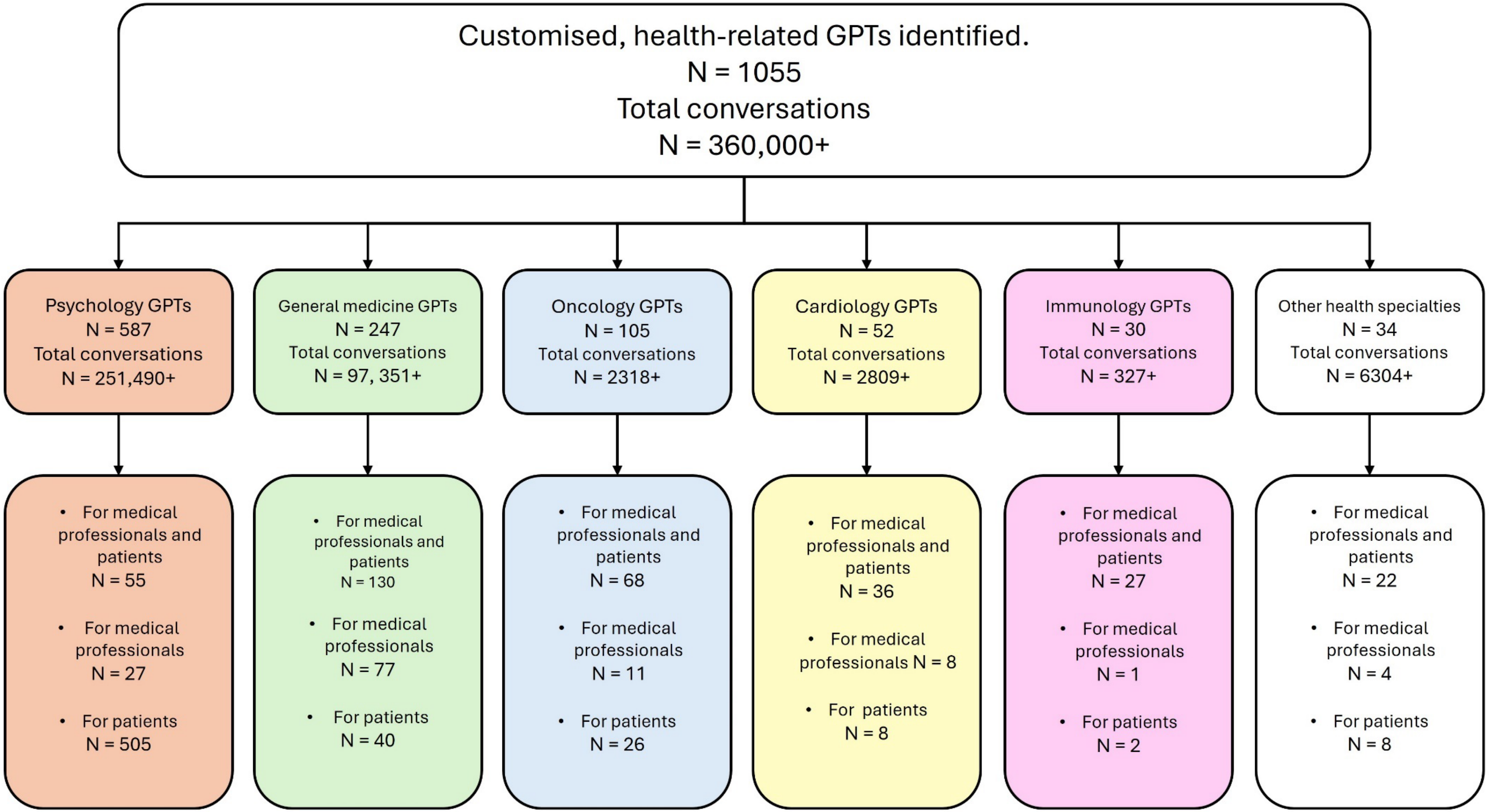

Supplement: Supplementary file 1 [file Supplementary_file_1.pdf]
